# Cas9 nickase-mediated contractions of CAG/CTG repeats are transcription-dependent and replication-independent

**DOI:** 10.1093/narmme/ugae013

**Published:** 2024-09-23

**Authors:** Meghan Larin, Florence Gidney, Lorène Aeschbach, Laura Heraty, Emma L Randall, Aeverie E R Heuchan, Marcela Buřičová, Melvin Bérard, Vincent Dion

**Affiliations:** UK Dementia Research Institute at Cardiff University, Hadyn Ellis Building, Maindy Road, Cardiff CF24 4HQ, UK; UK Dementia Research Institute at Cardiff University, Hadyn Ellis Building, Maindy Road, Cardiff CF24 4HQ, UK; Centre for Integrative Genomics, University of Lausanne, Lausanne CH-1015, Switzerland; UK Dementia Research Institute at Cardiff University, Hadyn Ellis Building, Maindy Road, Cardiff CF24 4HQ, UK; UK Dementia Research Institute at Cardiff University, Hadyn Ellis Building, Maindy Road, Cardiff CF24 4HQ, UK; UK Dementia Research Institute at Cardiff University, Hadyn Ellis Building, Maindy Road, Cardiff CF24 4HQ, UK; UK Dementia Research Institute at Cardiff University, Hadyn Ellis Building, Maindy Road, Cardiff CF24 4HQ, UK; Centre for Integrative Genomics, University of Lausanne, Lausanne CH-1015, Switzerland; UK Dementia Research Institute at Cardiff University, Hadyn Ellis Building, Maindy Road, Cardiff CF24 4HQ, UK; Division of Psychological Medicine and Clinical Neurosciences, Cardiff University, Maindy Road, Cardiff CF24 4HQ, UK

## Abstract

There is currently no disease-modifying treatment for expanded CAG/CTG repeat disorders. Given that longer repeat tracts lead to an earlier age of disease onset and faster progression, contracting them is expected to improve symptoms and/or delay onset. We have previously demonstrated that the Cas9 D10A nickase can effectively contract CAG/CTG repeats when targeted to the repeat tract itself. However, the mechanism remains unclear. Here, we tested whether nickase-mediated contractions depend on transcription or on replication using human cell models. We find that transcription promotes contractions and that they occur independently of the rate of cell division. These results support the therapeutic potential of this approach in non-dividing cells.

## Graphical Abstract

**Graphical Abstract:**
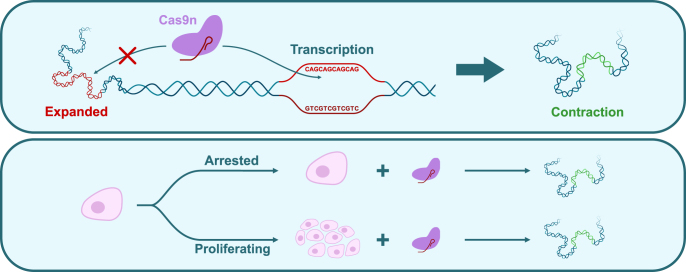

## Introduction

The expansion of CAG/CTG repeats causes at least 15 different neurodegenerative and neuromuscular disorders, including Huntington's disease (HD), myotonic dystrophy type 1 (DM1), and several spinocerebellar ataxias (1). There are currently no disease-modifying treatments available for any of these disorders. Importantly, in HD, repeat tract length accounts for about 60% of the variation in the age at disease onset, with longer repeats leading to an earlier appearance of the symptoms (2). This inverse correlation between repeat size and age at disease onset is also apparent in several other expanded CAG/CTG repeat disorders, suggesting that reducing the rate of expansion would slow the progression, if not prevent it in multiple diseases (3). This model is further substantiated by the identification of the mismatch repair pathway as a major modifier of the age at disease onset (4–6). Indeed, mismatch repair has a central role in the rate of repeat expansion in somatic tissues (7), providing a potential link to disease progression. Furthermore, contracting the repeat tract may be more effective at provoking a phenotypic change than merely preventing somatic expansions (8).

To date, three methods for contracting CAG/CTG repeats have been used (9–12). One is via a small molecule, naphthyridine-azaquinolone (NA), which binds to secondary structures formed by CAG/CTG repeats (10). NA induced contractions in HD-derived fibroblasts, HT1080 cells containing 850 CAG/CTG repeats at an ectopic locus, and in two mouse models (10,13). The current hypothesis to account for NA promoting contractions is centred on its ability to stabilize secondary DNA structures produced by transcription through the repeat tract, which are then processed by the mismatch repair complex MutSβ (10). These data suggest that contractions in this case occur via a mechanism related to that of somatic expansion.

A second approach is to induce double-strand breaks within the repeat tract with programmable nucleases, including zinc-finger nucleases (9,11), TALENs (12) and CRISPR-Cas9^9^. In these cases, the double-strand break(s) created within the repeat are thought to be repaired via homology directed repair, either in a Rad51-dependent manner (11), or through single-strand annealing (12). In our previous study, we found that both zinc-finger nucleases and CRISPR-Cas9 induced expansions and contractions at comparable rates (9). The risk, therefore, is that some cells within a population show further expansions, which would exacerbate disease phenotypes.

We have taken a third approach that avoids double-strand break-inducing nucleases. Instead, we used the Cas9 D10A nickase. When directed to the repeat tract itself using a single-guide RNA (sgRNA), we found that the Cas9 D10A nickase induces contractions efficiently in HEK293-derived cells (9), in astrocytes and neurons from patient-derived induced pluripotent stem cells (14), as well as in a mouse model of HD (14). We found that nickase-mediated contractions occurred only at expanded alleles larger than 41 repeats, and that off-target mutations, if present, were below detection limits (9,14). How contractions occur, however, is unclear. In a HEK293-derived model, contractions were unaffected by the knockdown of MSH2, a central player in mismatch repair (9). This suggests that the mechanism of contractions caused by the Cas9 nickase may be different to that of NA- and programmable nuclease-induced contractions.

Understanding how the Cas9 nickase induces contractions is critical for the translation of this approach into the clinic. Here we address two outstanding mechanistic questions about Cas9 nickase-mediated contractions: (i) are Cas9 nickase-mediated contractions dependent on transcription through the repeat tract? and, (ii) are they driven by replication? Our work indicates that contractions are promoted by transcription through the repeat tract while being uncoupled from replication rates. These results support the use of the Cas9 nickase as a therapy as contractions will be expected in non-dividing neurons and other terminally differentiated cells that express the mutant gene.

## Materials and methods

### Cell lines and culture conditions

The cell lines used in this study and their construction are found in Table S1. GFP(CAG)_101_^15^ cells were maintained at 37°C with 5% CO_2_ in Dulbecco's modified Eagle's medium (DMEM) glutamax, supplemented with 10% dialysed foetal bovine serum (FBS, Gibco), 100 U ml^−1^ penicillin, 100 μg ml^−1^ streptomycin, 15 μg ml^−1^ blasticidin and 150 μg ml^−1^ hygromycin. GNickx and GNickSx cells were cultured under the same conditions, but with the addition of G418 (400 μg ml^−1^) and puromycin (1 μg ml^−1^). The FLAH25 cells (15) were maintained at 37°C with 5% CO_2_ in DMEM F-12, 10% FBS (Gibco), 100 U ml^−1^ penicillin, 100 μg ml^−1^ streptomycin, G418 (500 μg ml^−1^) and puromycin (2.5 μg ml^−1^). FNick102 cells, which stably express Cas9 D10A together with the blasticidin resistance gene (BSD), were further supplemented with 10 μg ml^−1^ blasticidin. Cells were arrested using 0.5 μM palbociclib. The media was refreshed every 2–3 days.

Both GFP(CAG)_101_ and FLAH25 cells were genotyped using the Cell Line Authentication service from Eurofins. They were confirmed to be HEK293.2sus and HT1080, respectively. Mycoplasma testing was performed by Eurofins and using in-house testing regularly throughout the study and were found to be negative. The repeat tract length for each cell line was determined using PCR for the region around the repeat tract and Sanger sequencing or long-read sequencing. The primers used for PCR are found in Table S2.

Transient transfections of GFP(CAG)_101_ cells were conducted as described (16). GNickX and GNickSX cells were cultured for up to 42 days in the presence or absence of 2 μg ml^−1^ of doxycycline (dox). Cells were collected and genomic DNA was used to amplify the repeat tract using primers oVIN-0016 and oVIN-0487 (See Table S2). The plasmids used are found in Table S3. Specifically, lentiCas9n(D10A)-Blast (17) was a gift from Feng Zhang (Addgene plasmid # 63593) and hCas9_D10A (18) was a gift from George Church (Addgene plasmid # 41816).

### Cell sorting

FNick102 cells were transduced at a multiplicity of infection of 5 with viruses containing sgCTG and a GFP cassette (Table S3). Two days after transduction, cells were treated with or without doxycycline. At the end of the 21- or 42-day treatment, the cells were harvested with PBS + 10mM EDTA and sorted using a BD FACSAria II cell sorter based on their GFP intensity. Cells that had GFP fluorescence comparable to that of untransduced cells were kept and analysed (GFP^–^). Cells that were above the background fluorescence were further divided into two populations with the bottom 50% of GFP-expressing cells being referred to as GFP^+^ and the top 50% as GFP^++^.

### Small pool PCR

Small pool PCR was performed as before (19) with the following exceptions. Cells were harvested 3 weeks or 6 weeks after doxycycline addition, and DNA was extracted and then quantified using the Quant-iT Qubit dsDNA HS Assay Kit (Fisher Scientific). Blinded counts were completed for each membrane based on allele frequency calculated using a Poisson distribution. Differences between conditions were determined statistically by Mann–Whitney *U* test using GraphPad Prism software (v10.2.2).

### Long read sequencing

Single-molecule real time (SMRT) sequencing was done using an amplicon-based approach. Briefly, the repeat region in GNickS12, GNickS115 and GNick102 were amplified using primers containing the barcodes (see Table S2) together with Thermo™ Phusion II High Fidelity polymerase. Multiple independent PCRs were performed for each sample and pooled together before loading. Equimolar pools of the samples were loaded at a concentration between 10 and 12 pM. The sequencing was done using a Sequel IIe at Cardiff University School of Medicine. Repeat size was determined from the resulting CCS using Repeat Detector v1.0 as described (20).

### BrdU incorporation and cell cycle analysis

For BrdU staining, FLAH25 cells had their medium supplemented with BrdU (10 μg ml^−1^) for 1 h before being fixed, stained and imaged. The protocol used was as described (14) except that DNA was denatured with 2M HCl, followed by neutralization with 0.1M sodium borate. Cells were stained using the BU1/75 (ICR1) antibody (Abcam) with a dilution of 1:1000 and counterstained with DAPI. The images were taken using an Opera Phenix and analysed using FIJI (21) to count the total number of nuclei per frame. Cell cycle analysis was done using a 5-minute cell cycle assay from Chemotec and measured using a NucleoCounter NC-250 Automated Cell Analyzer. The data was then analysed using FlowJo (v10.0.8r1).

## Results

### Transcription through the repeat tract promotes Cas9 nickase-mediated contractions

**Figure 1.**
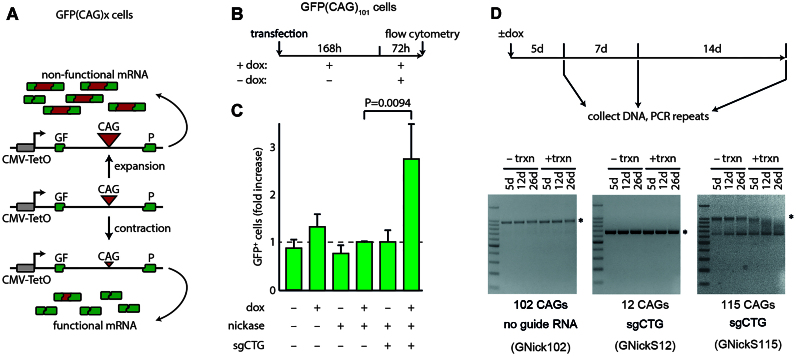
Transcription is essential for Cas9 nickase-mediated contractions in HEK293-derived cells. (**A**) Schematic of the GFP minigene reporter. Longer repeats are spliced into the mRNA along with 38 nucleotide downstream of the repeat tract, creating a frameshift and the degradation of the mRNA (42). Conversely, shorter repeats allow for efficient splicing of the reporter and robust GFP expression. The promoter, CMV-TetO, is induced by doxycycline (dox). (**B**) Timeline of experiments. (**C**) Fold change of GFP^+^ cells, which are enriched for contractions, over the control condition where dox is added and the Cas9 nickase is transfected, but there is no sgRNA expression. *N* ≥ 4 for each condition. (**D**) Timeline of experiments (top) for stable GNick and GNickS cells. After 5, 12 and 26 days of doxycycline addition, the cells were harvested, and a PCR of the repeat region was run on agarose gels. The asterisk indicates the modal repeat size in each line at the start of the experiment.

To address whether transcription promotes contractions, we used HEK293-derived GFP(CAG)_101_ cells (9,16,22). This line contains a doxycycline-inducible promoter driving transcription through 101 CAG repeats located in the intron of a GFP mini gene (Figure 1A). The repeats interfere with splicing in a length-dependent manner, with longer repeats resulting in lower GFP expression and shorter repeats increasing GFP levels, providing a fluorescent proxy for repeat size (9,22). This line has a single copy of the GFP minigene integrated on chromosome 12 (23). It recapitulates the instability pattern seen in other systems, including an expansion bias and HDAC3-dependency (24–28).

To determine whether transcription through the repeat tract promotes contractions, we transiently transfected the Cas9 nickase together with either a sgRNA against the repeat tract (sgCTG) or an empty vector. We either induced transcription through the repeat tract by adding doxycycline to the cultures for 168 h or kept them without dox and thus without transcription (Figure 1B). We then added doxycycline to all cultures for 72 h to read out steady state GFP levels. We found that transfection of both the Cas9 nickase and the sgCTG led to a significant increase in the GFP^+^ cells in the presence of doxycycline compared to the control conditions transfected with an empty plasmid and without dox (*P* = 0.0094, using a Kruskal–Wallis test with Dunn's multiple comparison post-hoc test). The GFP^+^ population is enriched with contractions (9) and contains cells with GFP intensities that lie within the brightest 1% of the cells transfected with the empty sgRNA vector, which is the negative control in these experiments (16). Importantly, we found no significant increase in GFP^+^ cells in the absence of doxycycline (*P* = 1.0), sgCTG (*P* = 1.0), or the nickase (*P* = 0.39; Figure 1C), suggesting that transcription is essential for contractions in HEK293-derived cells.

### Production of stable cell lines constitutively expressing the Cas9 nickase and sgCTG

Our previous results suggested that non-expanded (≤42) repeats are not targeted for contraction (9,14). This observation, together with the result that transcription is essential for Cas9 nickase-mediated contractions, suggested that we could construct a cell line that stably and continuously expresses both the Cas9 nickase and the sgCTG. This cell line should be viable and only contract the ectopic repeat when there is transcription through the repeat tract. To do this, we constructed GFP(CAG)_115_ cells that constitutively express the Cas9 nickase together with sgCTG (GNickS115, Table S1). The addition of doxycycline to these cells led to contractions, including in the non-expanded range as measured by PCR (Figure 1D). By contrast, control lines containing 102 CAGs and an empty sgRNA vector (GNick102) or lines with a non-pathogenic 12 CAGs (GNickS12) both remained stable. We noted that there is a low molecular band that appears in the GNickS115 lines, which is not seen in the absence of the sgRNA (GNick102) or when the repeat tract is short (GNickS12). We wondered whether this was a contraction of the expanded repeat tract in a fraction of the cells in the clone containing the sgRNA, the Cas9 nickase, and the large repeat. To address this, we used Single-Molecule Real-Time (SMRT) long-read sequencing on the same experiments and found that indeed they were contracted alleles. Further, we confirmed that contractions accumulated in GNicKS115 at a higher frequency at day 26 when the cells were treated with dox. We found that the main contracted band seen on the gel had a mode of three CAG repeats with intact flanking sequences (Supplementary Figure S1). Together, these results show that transcription promotes nickase-mediated contractions and emphasises that constitutive expression of the Cas9 nickase is not toxic despite the presence of endogenous, non-expanded repeats.

**Figure 2.**
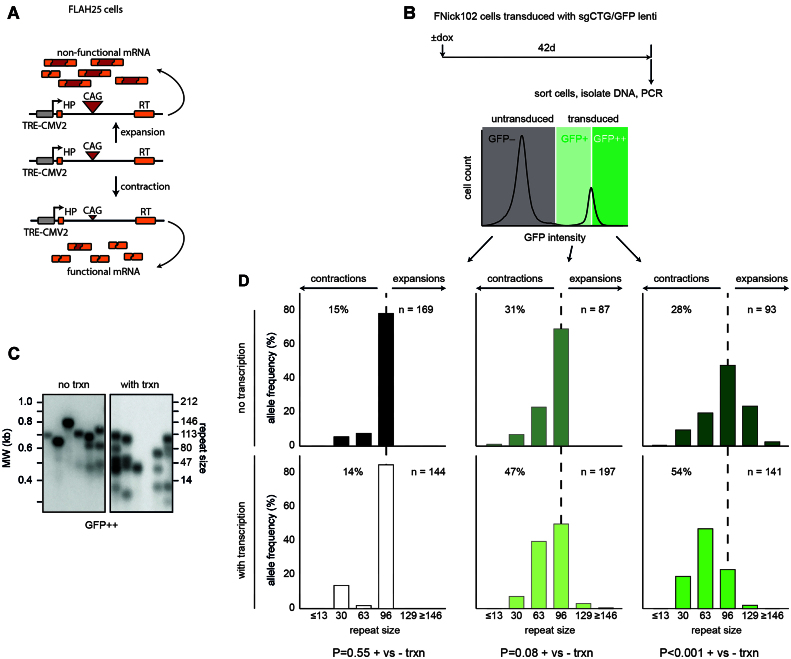
Transcription promotes nickase-mediated CAG repeat contraction in HT1080-derived cells. (**A**) FLAH25 cells contain a doxycycline inducible promoter driving transcription through an *HPRT*minigene in which 102 CAG repeats were inserted. As in the GFP reporter in GNickS lines, the presence of the repeats interferes with *HPRT* splicing. Here, however, we did not use the reporter. (**B**) FNick102 cells were transduced with a lentivirus that expresses both sgCTG and GFP and allowed to proliferate for 6 weeks. Then the cells were sorted into GFP^+^ cells, which are the dimmest 50% of the GFP-expressing cells, GFP^++^ cells, which express the largest amount of GFP, or GFP^–^ cells, which did not express GFP above background at the end of the experiments. All three populations are grown in the same well. (**C**) Representative small pool PCR of GFP^++^ FNickS102 cells treated with or without doxycycline. (**D**) Quantification of the GFP^+^, GFP^++^ and GFP^–^ cells in the presence or absence of doxycycline. The % in each graph represents the fraction of contractions. The number of repeats on the x-axes correspond to the median repeat size of a given interval, except for the ≤13 and ≥146 intervals. The *P*-values were calculated with Mann–Whitney *U* test between the GFP^+^ cells with or without doxycycline.

To test whether this effect of transcription is unique to HEK293-derived cells, we used a separate human cell line: the HT1080-derived FLAH25 (29). HT1080 cells have been used extensively to investigate repeat instability (11,15,29–33), making them a convenient model. Similar to GFP(CAG)_101_, FLAH25 cells harbour 102 CAGs in the intron of an ectopic *HPRT* minigene under the control of doxycycline (15). We transduced these cells with a lentivirus expressing the Cas9 nickase and isolated single clones to create a stable FLAH25 Nickase with 102 CAGs (FNick102 – Table S1). We then delivered the sgCTG using a lentiviral vector that also contained a GFP cassette, generating FNickS102 cells (Figure 2A). Unlike the GNickS115 cells, FNickS102 cells are a population with the sgCTG/GFP lentivirus delivered *de novo* for each experiment. We incubated FNickS102 cells with or without doxycycline and then sorted the GFP negative cells and the dimmest (GFP^+^) and brightest (GFP^++^) halves of the GFP-expressing cells for DNA extraction and analysis by small-pool PCR (34) (Figure 2B, C). The expectation was that cells with a large amount of GFP also express more sgCTG but the transcription through the repeat tract in the *HPRT* minigene would remain the same between the two populations. GFP^+^ cells did not show a statistically significant shift when comparing with and without doxycycline treatment at day 42 (Figure 2D – *P* = 0.08). We also found no significant increase in contraction in the GFP^++^ cells exposed to doxycycline after 21 days compared to those without doxycycline (Supplementary Figure S2 – *P* = 0.44). However, there was a clear effect of doxycycline treatment in increasing contractions after 42 days compared to cells cultured without doxycycline (Figure 2D – *P*< 0.0001 for GFP^++^ cells). These results suggest that, like in the GNickS115 cells, transcription through the repeat tract promotes contractions in HT1080-derived cells.

### Cas9-mediated contractions occur independently of proliferation rates

**Figure 3.**
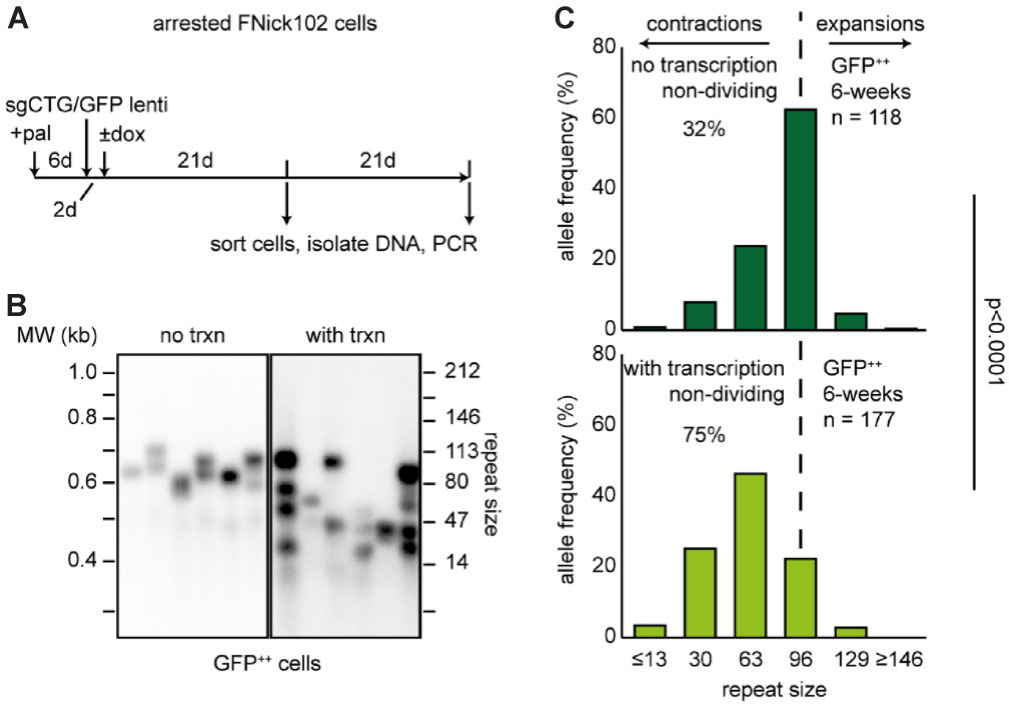
Transcription promotes nickase-mediated CAG repeat contraction independently of cell proliferation. (**A**) Timeline of the experiments (pal = palbociclib, dox = doxycycline). (**B**) Representative small pool PCR of GFP^++^ cells after 42 days. (**C**) Quantification of the small pool PCRs for GFP^++^ cells at 42 days in culture. The % in each graph represents the fraction of contractions. The number of repeats on the x-axes correspond to the median repeat size of a given interval, except for the ≤13 and ≥146 intervals. The *P*-value was calculated with Mann–Whitney *U* test between the GFP^++^ cells with or without doxycycline.

One advantage of the HT1080 cells is that they can be efficiently arrested using the CDK4/6 inhibitor palbociclib (35). This allowed us to test whether contractions occur in cells whose proliferation is compromised, modelling disease-relevant cells, such as neurons, that are terminally differentiated. We found that FLAH25 cell counts plateaued over 6 days of palbociclib treatment (Supplementary Figure S3A). The cells arrested as measured by the lack of BrdU incorporation and DAPI staining (Supplementary Figure S3A). Given these observations, we arrested FNick102 cells using palbociclib 6 days prior to transduction with the sgCTG/GFP lentivirus. Two days after transduction, we induced transcription through the repeat tract by adding doxycycline to the culture media (Figure 3A). As before, we sorted cells based on the GFP expression at 21 and 42 days and performed small-pool PCR to determine repeat size distribution (Figure 3B, Supplementary Figure S3B). We found that contractions occurred readily in palbociclib-arrested cells (Figure 3B, C, Supplementary Figure S3C), suggesting that population doubling does not correlate with frequencies of nickase-mediated contractions.

To test whether contractions in palbociclib-arrested cells are also dependent on transcription, we compared cells arrested both with and without doxycycline treatment. We found that, similar to replicating cells, palbociclib-arrested FNickS102 cells showed enhanced contraction frequencies when exposed to doxycycline after 21 and 42 days (Supplementary Figure S3C; Figure 3C, *P* < 0.0001), suggesting that transcription through the repeat tract promotes nickase-mediated contractions in non-replicating FNickS102 cells.

## Discussion

Here we found that transcription promotes contractions when the Cas9 D10A nickase is paired with a sgRNA against CAG repeats in two independent cell lines. Moreover, nickase-induced contractions did not scale with the number of cell divisions, suggesting that contractions can occur without a major role for replication. This adds to our previous work showing that the same treatment induced contractions in patient-derived induced pluripotent stem cells differentiated into astrocytes and neurons (14).

In FNickS102 and GNickS115 cells, we found that simply culturing the cells without doxycycline for 6 weeks led to an increase in contractions compare to cells that were untransduced (Figures 1D and 2D). We found previously that in GFP(CAG)x cells, there is some transcription through the locus in the absence of doxycycline as evidenced by low but detectable background GFP expression (9). We speculate that this leaky transcription may be responsible for the contractions observed under these conditions. Another potential model is that the large amount of sgCTG masks any effect of transcription, perhaps because the sgCTG is rate-limiting. This is supported by the population of GFP^+^ FNickS102 cells, which contains less of the sgCTG/GFP lentivirus, yielding lower levels of contractions compared to the GFP^++^ population (Figure 2D). The prediction is that transducing FNick102 cells with a larger amount of sgCTG may lead to further contractions even in the absence of transcription. It is also unclear why there are no significant contractions until 6 weeks in the FNickS102 GFP^++^ cells. The simplest explanation for this lag is that contractions are slow to accumulate and do not reach significance within 3 weeks. A non-mutually exclusive hypothesis is that the contractions occur via small changes in repeat size that we cannot resolve on the small-pool PCR gel after only 3 weeks. Regardless of the exact reason for these results, our conclusion still holds: transcription through the repeat tract promotes Cas9 nickase-mediated contractions.

Transcription has been shown to promote spontaneous and NA-mediated contractions in HT1080-derived cells (10,15). In these cases, the prevailing model is that transcription produces R-loops that are then processed, possibly via the mismatch repair pathway, to lead to contractions (10,30). In the case of the Cas9 nickase, there are further possibilities. For example, Cas9 is inhibited by the presence of nucleosomes (36,37). Indeed, CAG/CTG repeat tracts are strong nucleosome-positioning sequences (38–41) and transcription may act in displacing nucleosomes such that Cas9 can access its target sequence. This would also be in line with our previous work, which showed that knockdown of MSH2 and XPA, essential for mismatch repair and transcription-coupled nucleotide excision repair, respectively, had no effect on Cas9 nickase-induced contractions (9). Another possibility is that the R-loop is provided by Cas9/sgRNA binding to DNA.

We have developed two different tools that will allow us to probe the mechanism of nickase-induced contractions further. Specifically, we have created stable cell lines that constitutively express both the Cas9 nickase and the sgCTG. Given the ease of genetic engineering in immortalized cell lines, it will be possible to make genetic modifications to test specific models of repeat contractions. Consistent with our previous findings that short (≤42) repeats do not appear to be mutated by the Cas9 nicakse (9,14), the viability of these stable lines suggests that there is little overt toxicity caused by the Cas9 nickase even in the presence of a sgRNA against the repeat tract. The results presented here help further demonstrate that the Cas9 nickase constitutes a potential therapeutic intervention for expanded repeat disorders. Continuing to elucidate the underlying mechanism will help improve both the efficiency and safety of the approach.

## Supplementary Material

ugae013_Supplemental_File

## Data Availability

All cell lines and raw data generated herein are available upon request from the corresponding author. The sequencing data is available publicly via Sequence Read Archive (SRA) (ID: PRJNA1153810) - https://www.ncbi.nlm.nih.gov/bioproject/1153810.

## Notes

Present address: Meghan Larin, AstraZeneca R&D, 1 Francis Crick Avenue, Cambridge CB2 0AA, UK.

Present address: Florence Gidney, Huntington's Disease Centre, Department of Neurodegenerative Disease, UCL Queen Square Institute of Neurology, University College London, London WC1N 3BG, UK.

Present address: Lorène Aeschbach, AC Immune SA EPFL, Innovation Park Building B, 1015 Lausanne, Switzerland.

Present address: Marcela Buřičová, Revvity, Cambridge Research Park, 8100 Beach Dr, Waterbeach, Cambridge CB259TL, UK.

Present address: Melvin Bérard, Department of Cellular and Molecular Biology, University of Geneva, Quai Ansermet 30, 1205 Geneva, Switzerland.
