# Supplementary material for: Cas9 nickase-mediated contractions of CAG/CTG repeats are transcription-dependent and replication-independent
Source: NAR Mol Med. 2024 Sep 23;1(4):ugae013. doi: 10.1093/narmme/ugae013 (PMC12429965; doi:10.1093/narmme/ugae013)
Supplement: ugae013_Supplemental_File [file ugae013_Supplemental_File.pdf]

Supplementary information

Cas9 Nickase-mediated contractions of CAG/CTG repeats are transcription-dependent and replication-independent

Meghan Larin[1,2], Florence Gidney[1,3], Lorène Aeschbach[4,5], Laura Heraty[1], Emma Randall[1], Aeverie E. R. Heuchan[1], Marcela Buřičová[1,6], Melvin Bérard[4,7], and Vincent Dion[1,8,9]

1: UK Dementia Research Institute at Cardiff University, Hadyn Ellis Building, Maindy Road, Cardiff, UK, CF24 4HQ.

2: Current address: AstraZeneca R&D 1 Francis Crick Avenue Cambridge, UK CB2 0AA

3: Current address: Huntington's Disease Centre, Department of Neurodegenerative Disease, UCL Queen Square Institute of Neurology, University College London, London WC1N 3BG, UK

4: Centre for Integrative Genomics, University of Lausanne, Lausanne, Switzerland, CH-1015

5: Current address: AC Immune SA EPFL Innovation Park Building B 1015 Lausanne, Switzerland

6: Revvity, Cambridge Research Park, 8100 Beach Dr, Waterbeach, Cambridge, CB259TL

7: Current address: Department of Cellular and Molecular Biology, University of Geneva, Quai Ansermet 30, 1205 Geneva.

8: Division of Psychological Medicine and Clinical Neurosciences, Cardiff University, Maindy Road, Cardiff, UK, CF24 4HQ

9: Corresponding author, dionv@cardiff.ac.uk

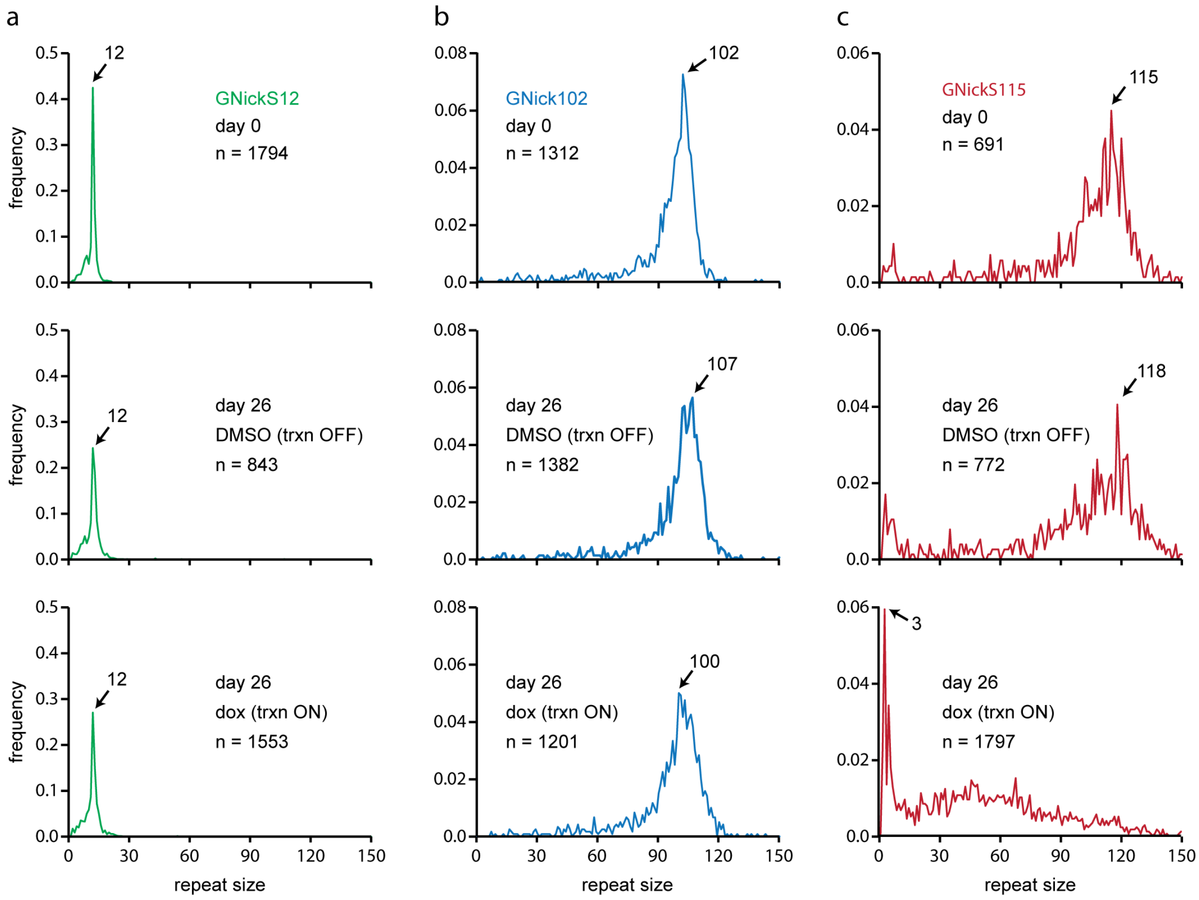

Suppl Figure 1: *Long read sequencing confirms contractions in GNickS115. A) GNickS12 cells containing 12 CAG repeats and expressing both the Cas9 nickase and sgCTG before treatment (top), after 26 days in DMSO (transcription OFF – middle) or doxycycline (transcription ON – Bottom). B) GNick102 cells containing 102 CAG repeats and expressing the Cas9 nickase but not the sgCTG before treatment (top), after 26 days in DMSO (transcription OFF – middle) or doxycycline (transcription ON – Bottom). C) GNickS115 cells containing 115 CAG repeats and expressing both the Cas9 nickase and sgCTG before treatment (top), after 26 days in DMSO (transcription OFF – middle) or doxycycline (transcription ON – Bottom). In all panels, the n is the number of reads in the condition shown. The modal repeat size for each condition is shown above the distribution with the arrow. These samples are the same as the ones shown in Panel 1D.*

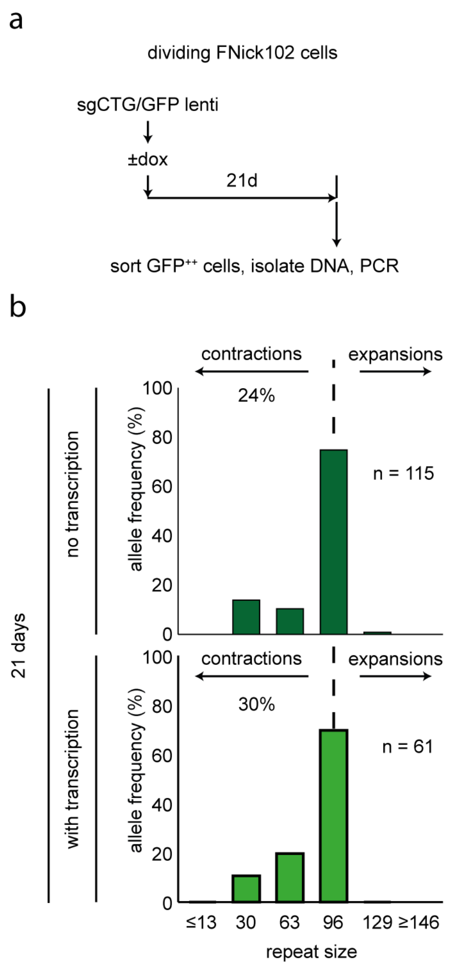

Suppl Figure 2: *Cas9-mediated contractions in dividing FNickS102 cells after 21 days with or without transcription. A) timeline of the experiments. B) Quantification of small-pool PCR blots after three weeks with or without doxycycline. This was done as in Fig. 2.*

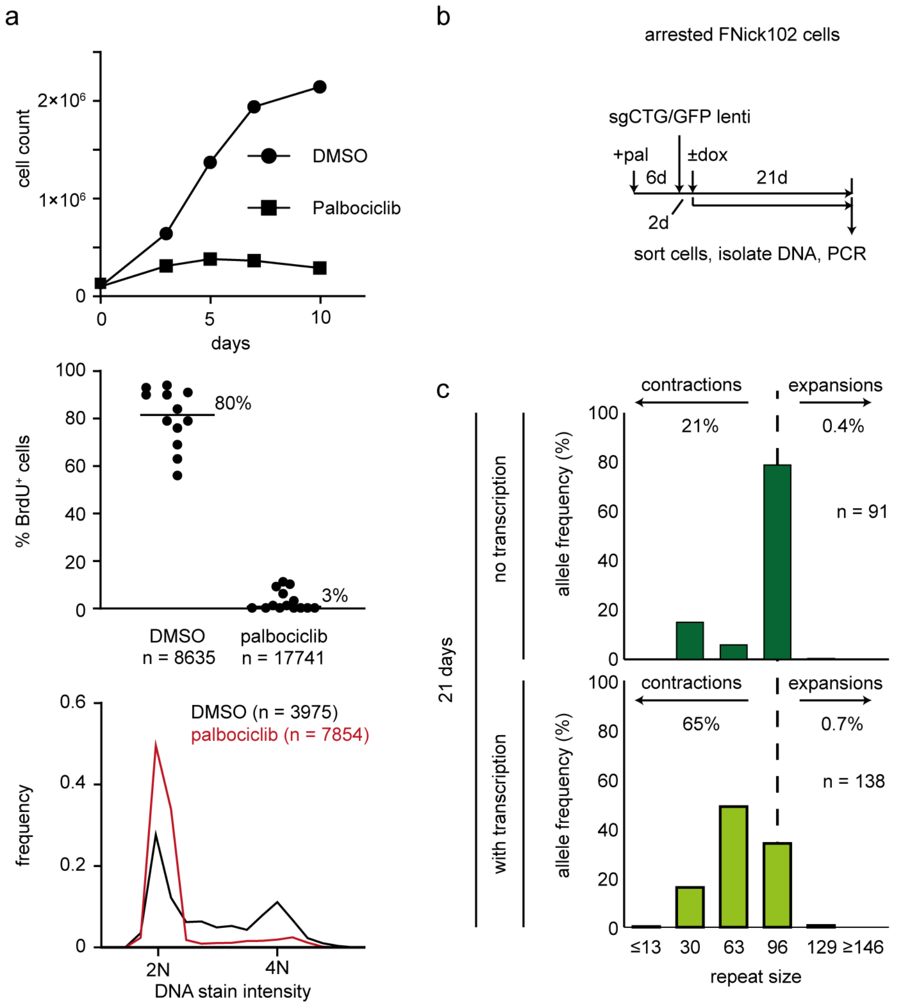

Suppl Figure 3: *Cas9-mediated contractions in arrested FNickS102 cells after 21 days. A) Top: Effect of palbociclib arrest on cell counts. Each point represents the average of 2 replicates. Middle: number of BrdU⁺ cells after one week of palbociclib treatment or its vehicle (DMSO). Each point is the % of BrdU⁺ in a field of view. The total number of cells counted is indicated below the axis. Bottom: Representative cell cycle profile for one of 3 populations tested in the presence of palbociclib (red) or DMSO only (black) after 7 days. The number of cells within this sample is indicated in the legend. B) timeline of the experiments for repeat contraction. C) Quantification of small-pool PCR blots after three weeks of palbociclib treatment with or without doxycycline. This was done as in Fig. 3 with GFP⁺⁺ cells.*

Table S1: Cell lines

Name	Parent	Transgene over parent	Plasmids used	Integration method	Resistance markers	Reference
T-REX Flp-in	HEK293.2sus	pFRT/*lac*Zeo<br>Tetracycline Repressor	-<br>pcDNA6/TR	-<br>-	Blasticidin<br>Zeocin	Thermo Fisher
CAS9NICK	T-Rex Flp in	Cas9 D10A	pCDNA3.3 – Cas9_D10A	Random	Blasticidin<br>Zeocin<br>G418	This study
CAS9NickGFP$_{98}$	CAS9NICK	GFP(CAG)$_{101}$	pBY-018	Flp-in mediated	Blasticidin<br>G418<br>Hygromycin	This study
GNickSX	CAS9NickGFP$_{98}$	sgCTG	pPN10-gCTG	Random	Blasticidin<br>G418<br>Hygromycin<br>Puromycin	This study
FLAH25	HT1080	Tetracycline Repressor<br>HPRT(CAG)$_{102}$	pTet-ON<br>pYL168	Random	G418<br>Puromycin	[16]
FNick102	FLAH25	Cas9 D10A	pLenti-Cas9(D10A)-Blast	Lentiviral transduction	G418<br>Puromycin<br>Blasticidin	This study
GFP(CAG)$_{101}$	T-REX Flp-in	GFP(CAG)$_{101}$	pGFP(CAG)$_{101}$	Flp-in mediated	Hygromycin<br>Blasticidin	[9,15]

*: X means any repeat size isolated from the same parent line.

Table S2: **Primers used in this study**

Primer	Sequence*	Target locus	Ref
oVIN-0016	5' CCT ATG CAT GTA TAC TAT ATG C	HPRT mini gene	[16]
oVIN-0487	5' TAG TGT CTA TCA ATA GTG GAC TGG	HPRT mini gene	[16]
oVIN-0459	5' AAG AGC TTC CCT TTA CAC AAC G	GFP mini gene	[9]
oVIN-0460	5' TCT GCA AAT TCA GTG ATG C	GFP mini gene	[9]
oVIN-0100	5' AG CAG CAG CAG CAG CAG CAG CAG CAG CAG C	CAG/CTG repeats	[18]
oVIN-2515	5' *TCA GAC GAT GCG TCA T* AAG AGC TTC CCT TTA CAC AAC G	GFP mini gene – GNickS12 day 0	This study
oVIN-2516	5' *CTA TAC ATG ACT CTG C* AAG AGC TTC CCT TTA CAC AAC G	GFP mini gene – GNickS12, dox-treated for 26 day.	This study
oVIN-2517	5' *TAC TAG AGT AGC ACT C* AAG AGC TTC CCT TTA CAC AAC G	GFP mini gene – GNickS12, DMSO-treated for 26 day.	This study
oVIN-2518	5' *TGT GTA TCA GTA CAT* AAG AGC TTC CCT TTA CAC AAC G	GFP mini gene – GNickS115 day 0	This study
oVIN-2519	5' *ACA CGC ATG ACA CAC T* AAG AGC TTC CCT TTA CAC AAC G	GFP mini gene – GNickS115, DMSO-treated for 26 day.	This study
oVIN-2520	5' *GAT CTC TAC TAT ATG C* AAG AGC TTC CCT TTA CAC AAC G	GFP mini gene – GNickS115, dox-treated for 26 day.	This study
oVIN-2524	5' *GCG CGA TAC GAT GAC T* AAG AGC TTC CCT TTA CAC AAC G	GFP mini gene – GNick102 day 0	This study
oVIN-2525	5' *CGC GCT CAG CTG ATC G* AAG AGC TTC CCT TTA CAC AAC G	GFP mini gene – GNick102, DMSO-treated for 26 day.	This study
oVIN-2526	5' *GCG CAC GCA CTA CAG A* AAG AGC TTC CCT TTA CAC AAC G	GFP mini gene – GNick102, dox-treated for 26 day.	This study

**: italics indicate barcode sequences.*

Table S3: **Plasmids used in this study**

Name	Description	Addgene reference	Reference
pcDNA3.3-TOPO -Cas9_D10A	Expresses Cas9_D10A from a CMV promoter. Alson harbours a G418 resistance gene.	41816	[42]
pPN10	Empty gRNA vector with a Puromycin resistance.	114386	[9]
pPN10 – sgCTG	Expresses the sgRNA against $(CTG)_6C$ from a U6 promoter. Also contain a Puromycin resistance gene.	114385	[9]
pBY-018	Contains the GFP(CAG)100 construct along with an INT sequence and a hygromycin resistance gene for Flp-In integration.	N/A	[22]
pLenti-Cas9(D10A)-Blast	Expresses the Cas9 nickase together with a blasticidin resistance gene. Suitable for lentivirus packaging.	63593	[43]
pLV[gRNA]-EGFP-U6>{sgCTG}	Contains a eGFP gene along with a U6 promoter driving the sgCTG (target sequence: $(CUG)_6C$.	216732	[14]
